# Assessing Attitudes Toward Transcranial Magnetic Stimulation: Perspectives of ChatGPT and Psychiatrists

**DOI:** 10.1192/j.eurpsy.2026.10720

**Published:** 2026-07-31

**Authors:** S. Karakaya, E. S. Ahi Üstün, Y. Uzel, K. C. Can

**Affiliations:** 1 Psychiatry, Mamak State Hospital; 2 Psychiatry, Ankara University Faculty of Medicine, Ankara, Türkiye

## Abstract

**Introduction:**

Transcranial Magnetic Stimulation (TMS) is a safe, non-invasive neurostimulation technique increasingly used in psychiatry(Sierra P, *et al*. Psychiatric Q 2024;95:271–285).Despite the fact that clinicians’ decisions may be influenced by knowledge, practical challenges, expected outcomes, and cultural factors, these aspects are rarely investigated in neuromodulation research(Van der Groen O. Neuromodulation 2023;26:705–706). In addition to these factors, both patients and clinicians are increasingly turning to artificial intelligence to inform treatment decisions.

**Objectives:**

In light of the above,we aimed to assess both ChatGPT-4o’s attitude toward TMS and the knowledge and attitudes of psychiatrists in Türkiye regarding this treatment modality.

**Methods:**

Psychiatrists working in Türkiye were recruited using convenience sampling, and the study was conducted via Google Forms.Participants rated their TMS knowledge on five items, yielding a composite score(score range:3–17). To evaluate attitudes toward TMS, the authors developed 10 items(score range:5–50). Attitude questions were administered to both ChatGPT and the participants.Descriptive statistics were computed for sample characteristics. Group differences were examined using the Mann–Whitney U test.Correlational analyses were conducted with Spearman’s correlation, and multiple linear regression analysis was employed to identify predictors of prejudice scores.(The ethics committee approval number is I04-302-25)

**Table 1.** Multiple linear regression predicting prejudice scores Table 1. Long description.

Predictor	B(Unstandardized)	Standard error	β(Standardized)	t	p
Constant	23.43	1.59	-	14.77	<.001*
Neurostimulation training	-3.53	0.99	-.29	-3.57	<.001*
Knowledge level	-0.38	0.19	-.21	-2.02	.046
Presence of TMS	0.58	1.18	0.05	0.49	.626
Observation of TMS	-2.11	1.30	-.17	-1.62	.107

*R=0.448, R²=0.201, Adjusted R² =0.175; Std. Error of Estimate=5.339; ANOVA* F(4,125)=7.86, p<.001; *p < 0.05 considered significant.

**Results:**

A total of 130 psychiatrists participated (81 female,49 male). Of these, 50.8% were residents, 40.8% specialists, and 8.5% professors. Prejudice scores were negatively correlated with knowledge (ρ=–0.39, p<.001). Participants had a mean prejudice score of 17.33±5.88, while ChatGPT-4o had a score of 15. Participants with neuromodulation training had considerably lower prejudice scores (U=1106.5, Z=–4.342, p<.001), whereas knowledge scores did not differ significantly (p=0.32). Prejudice scores were lower in clinics with TMS access (50%) compared to those without (Prejudice:U=1212.5, Z=–4.203, p<.001). Multiple regression significantly predicted prejudice scores (F(4,125)=7.86, p<.001, R²=.20). Neurostimulation training and knowledge level were significant negative predictors (Table 1.).

**Conclusions:**

Training in neuromodulation and clinical use of TMS appear to reduce prejudice toward this treatment. ChatGPT’s lower bias score suggests that AI could complement clinical decision-making processes.

**Disclosure of Interest:**

None Declared

## Long descriptions

### Table 1. Long description

The table consists of six columns: Predictor, B (Unstandardized), Standard error, beta (Standardized), t, and p.

* Constant: B = 23.43, Standard error = 1.59, t = 14.77, p < .001.

* Neurostimulation training: B = -3.53, Standard error = 0.99, beta = -.29, t = -3.57, p < .001.

* Knowledge level: B = -0.38, Standard error = 0.19, beta = -.21, t = -2.02, p = .046.

* Presence of T M S: B = 0.58, Standard error = 1.18, beta = 0.05, t = 0.49, p = .626.

* Observation of T M S: B = -2.11, Standard error = 1.30, beta = -.17, t = -1.62, p = .107.

Model statistics provided below the table: R = 0.448, R-squared = 0.201, Adjusted R-squared = 0.175. Standard Error of Estimate = 5.339. ANOVA F (4, 125) = 7.86, p < .001. Asterisks indicate p < 0.05 is considered significant.

Navigate back to Table 1..

